# Regular-Type Liesegang Pattern of AgCl in a One-Dimensional System

**DOI:** 10.3390/ma14061526

**Published:** 2021-03-20

**Authors:** Shun Sakamoto, Masaki Itatani, Kanta Tsukada, Hideki Nabika

**Affiliations:** 1 Faculty of Science, Yamagata University, 1-4-12 Kojirakawa, Yamagata 990-8560, Japan; s16c017@st.yamagata-u.ac.jp; 2 Graduate School of Science and Engineering, Yamagata University, 1-4-12 Kojirakawa, Yamagata 990-8560, Japan; s192101d@st.yamagata-u.ac.jp (M.I.); s203018m@st.yamagata-u.ac.jp (K.T.)

**Keywords:** Liesegang, reaction–diffusion, non-linear, self-organization

## Abstract

The Liesegang phenomenon can be used for micro- and nanofabrication processes to yield materials with periodic precipitation of diverse types of materials. Although there have been several attempts to control the periodicity of the Liesegang patterns, it remains unclear whether the periodic precipitation of AgCl in gel medium causes regular- or revert-type patterns. To confirm the periodicity of the AgCl pattern, we conduct one-dimensional experiments under various ion concentration conditions. From microscopic observations, three different precipitation modes were observed, i.e., continuous precipitation with a sharp front, periodic precipitation and continuous precipitation with a gradual front. For these three modes, numerical analyses of the pattern geometry are performed for the periodic precipitation. It was confirmed that the regular-type pattern appeared for all concentration conditions conducted in the present experiments. Furthermore, the pattern was found to obey the spacing law and the Matalon–Packter law. From our experiments, we concluded that AgCl forms regular-type Liesegang patterns, regardless of the dimension of diffusion.

## 1. Introduction

The spontaneous formation of spatiotemporally periodic structures is an essential aspect that can be seen in nature and sometimes gives rise to a variety of functions in the field of micro- and nanotechnology [1]. The Liesegang pattern was found in 1896 by German chemist Raphael E. Liesegang, which is currently regarded as a spontaneous pattern formation in chemical systems. In a typical system, two reaction substrates (A and B) were initially dissolved into two independent media: A in an aqueous solution and B in a gel. Upon making contact with these two media, A in the aqueous solution (with a higher concentration) diffuses into the gel doped with B (with a lower concentration). During this diffusion process, A and B react to form C. Under some chemical and physical conditions, C precipitates periodically with repetitive precipitated and depleted zones. For example, Co(OH)_2_ precipitates periodically when NH_4_OH in the aqueous solution diffuses into the gel doped with CoCl_2_, according to the reaction CoCl_2_ + 2 NH_4_OH → Co(OH)_2_ + 2 NH_4_Cl. By changing the shape of the gel, it is possible to yield gel materials with periodic Co(OH)_2_ precipitates with one [2], two [3] and three dimensions [4]. In addition to Co(OH)_2_, gels with various materials include hydroxide salts [5,6], chromate and dichromate salts [7,8,9,10], phosphate salts [11,12], metal nanoparticles [13,14,15] and polymers [16,17].

Despite the wide variety of materials and dimensions, only simple models have been proposed so far, and these are generally divided into two models: pre-nucleation and post-nucleation models [18]. The former assumes that the precipitated zones are formed at the location where nucleation ignites when the concentration of C exceeds a certain concentration threshold. However, the latter model assumes that the precipitated and depleted zones are formed via thermodynamic instability that forms periodic high- and low-concentration regions, even from homogeneously distributed initial conditions. As elementary steps to yield periodically precipitated (high concentration) and depleted (low concentration) zones are different in these two models, they need to be chosen appropriately depending on the experimental system. However, regardless of the model on which the structure’s formation is based, we can observe a common empirical law in the developed structure. Let the contact interface between the media containing A and B be the origin of the reaction space axis *x*, and let *x_n_* and *w_n_* be the position where the *n*th precipitation band appears and the interband spacing (*w_n_ = x_n+_*_1_ *− x_n_*), respectively. The spacing law, which is an empirical law, predicts that *x_n_* varies with geometric series.

(1) $$\frac{x_{n+1}}{x_{n}}=1+p$$

where *p* is the spacing coefficient. The value of *p* strongly depends on the experimental conditions, especially the initial concentrations of A and B [19].

(2) $$p=F\left(b_{0}\right)+G\left(b_{0}\right)\frac{b_{0}}{a_{0}}$$

where *a*_0_ and *b*_0_ are the initial concentrations of A and B, respectively. Equation (2) is known as the Matalon–Packter law and has been confirmed to be valid in both experimental and simulation results [19,20].

The *x_n_* and *w_n_* determine the geometry of the periodic structure and thus the characteristics of the patterned materials. Thus, it is necessary to control them to yield the desired periodically patterned materials. It has been reported that there are three different geometries depending on the geometry, namely, the regular (*w_n+_*_1_/*w_n_* > 1), equidistant (*w_n+_*_1_/*w_n_* = 1) and revert patterns (*w_n+_*_1_/*w_n_* < 1) [21]. The regular pattern is the primary geometry that has been observed in both experiments and simulations, where the interband spacing *w_n_* increases as one moves farther from the interface between two media. However, the revert pattern has been observed only in a limited number of systems in which the interband spacing *w_n_* is characterized by a narrowing as the distance from the interface increases. Although the formation mechanism of the regular pattern has been well explained by existing models, it is not straightforward to clarify the origin of the revert pattern. However, it is important to control the geometry of Liesegang patterns to yield well-designed periodic tailor-made patterns with geometric series.

Materials that exhibit the revert pattern are mainly Pb [22,23,24,25,26,27] and Ag [22,28,29,30] salts. One of the proposed mechanisms to form the revert pattern in Pb and Ag systems is the preferential adsorption theory, in which the key factor that switches the pattern between regular and revert is the surface charge of colloidal particles, which are precursors of the precipitates [22,29]. For example, for the system with A = Ag^+^, B = I^−^ and C = AgI, the preferential adsorption of excess silver ions on the surface of AgI colloids results in the formation of positively charged colloidal particles, which peptize the particles dispersed in the gel phase. This peptizing effect imposes an additional barrier to aggregation and prevents precipitation. Because the concentration gradient of silver ions forms from *x* = 0, the barrier to the aggregation and precipitation decreases from *x* = 0, leading to the precipitated bands at increasingly closer distances, that is, revert Liesegang geometry. A similar revert geometry was observed for AgI [22,28,29] and AgBr [30] systems, whereas it has been reported that AgCl exhibited a regular geometry [31]. There appear to be two possibilities that could change the pattern geometry from the revert-type of AgI and AgBr to the regular-type of AgCl: (i) the difference in the concentration gradient of silver ions and (ii) differences in the preferential adsorption to the colloidal particles. Since the difference in the concentration gradient between one-dimensional (1D) and two-dimensional (2D) systems was reported to affect the pattern geometry [32], a study on regular-type AgCl indicated that option (i) is a possibility, that is, the pattern of regular-type AgCl was obtained with a 2D thin gel film, which was different from the revert-type AgI and AgBr obtained in 1D test tubes. As noted above, since the concentration gradient of silver ions is the key parameter, the difference in diffusion behavior could alter the peptizing effect to stabilize the colloidal sol. The study stated that, in contrast to laminar diffusion in 1D systems, comparatively more space for 2D diffusion leads to perfect balancing of attractive and repulsive forces that result in regular-type patterns [31]. In fact, another study confirmed that the spacing coefficients *p* are different between 1D and 2D systems [32]. However, there is still no direct evidence that 2D diffusion is the dominant cause of regular-type AgCl pattern formation. This issue can be clarified to carry out experiments on AgCl pattern formation in 1D systems.

For this purpose, we conducted pattern-formation experiments using 1D test tubes under various concentration conditions using an AgNO_3_ aqueous solution and gelatin gel doped with NaCl. To eliminate the effect of light, all experiments were performed in the dark, unless otherwise stated. The obtained pattern was analyzed with *x_n_* and *w_n_*, which is a measure that is employed to determine whether the pattern is regular or revert. An attempt was also made to confirm the validity of the Matalon–Packter law for the observed periodic structures.

## 2. Materials and Methods

Gelatin was purchased from Nacalai Tesque, Inc. (Kyoto, Japan), and AgNO_3_ and NaCl were purchased from Kanto Chemical Co., Inc. (Tokyo, Japan). All the reagents were used without further purification. Gelatin solution (5.0%) was prepared by dissolving the required amount of gelatin in purified water while stirring and heating at 75 °C for 25 min. NaCl was added to the gelatin solution to obtain the desired final concentration. Then, the mixed gelatin solution was poured into a glass test tube and stored in an incubator at 18 °C overnight to yield a gelatin gel matrix. A solution of AgNO_3_ at the desired concentration was then poured on top of the gel in the test tube. The samples were stored in an incubator at 18 °C for 14 days in the dark. After 14 days of reaction, the test tubes were removed from the incubator and observed using a digital camera EX-ZR850 (Casio Computer Co. Ltd., Tokyo, Japan) and optical microscope SZ-61 (Olympus, Tokyo, Japan).

## 3. Results and Discussion

**Figure 1:**
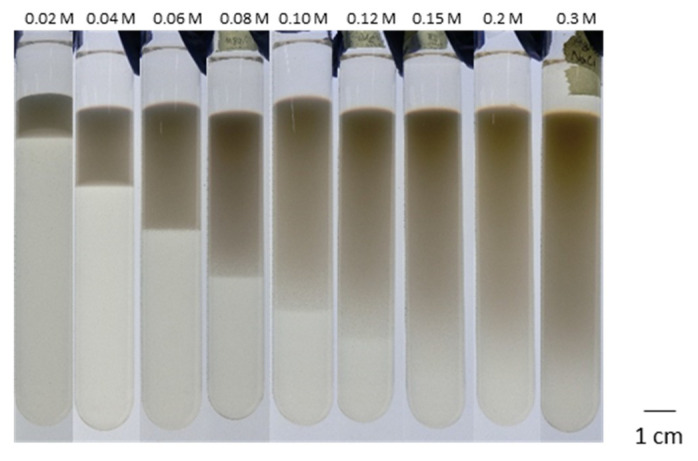
Formation of precipitated zone in a test tube. [NaCl] was fixed at 0.01 M. [AgNO_3_] varied from 0.02 to 0.3 M, as shown in each photo. The sample was incubated at 18 °C in the dark for 14 days.

We used a test tube to yield a 1D AgCl pattern in a gelatin gel medium. Typical experimental data are shown in Figure 1, where the photos were taken 14 days after pouring AgNO_3_ aqueous solution onto a gel doped with NaCl at 0.01 M. For [AgNO_3_] = 0.02 M, dark precipitates appeared below the solution–gel interface with a width of approximately 1 cm, where a sharp reaction front can be seen at the bottom of the precipitated zone. The width of the precipitated zone increased with increasing [AgNO_3_]. These results strongly indicate that the formation of the precipitated zone is governed by the diffusion of silver ions into the gel phase. For [AgNO_3_] = 0.10 M, the precipitated zone grew to more than 6 cm in width, and the reaction front became less sharp. A further increase in [AgNO_3_] made it impossible to define the reaction front.

**Figure 2:**
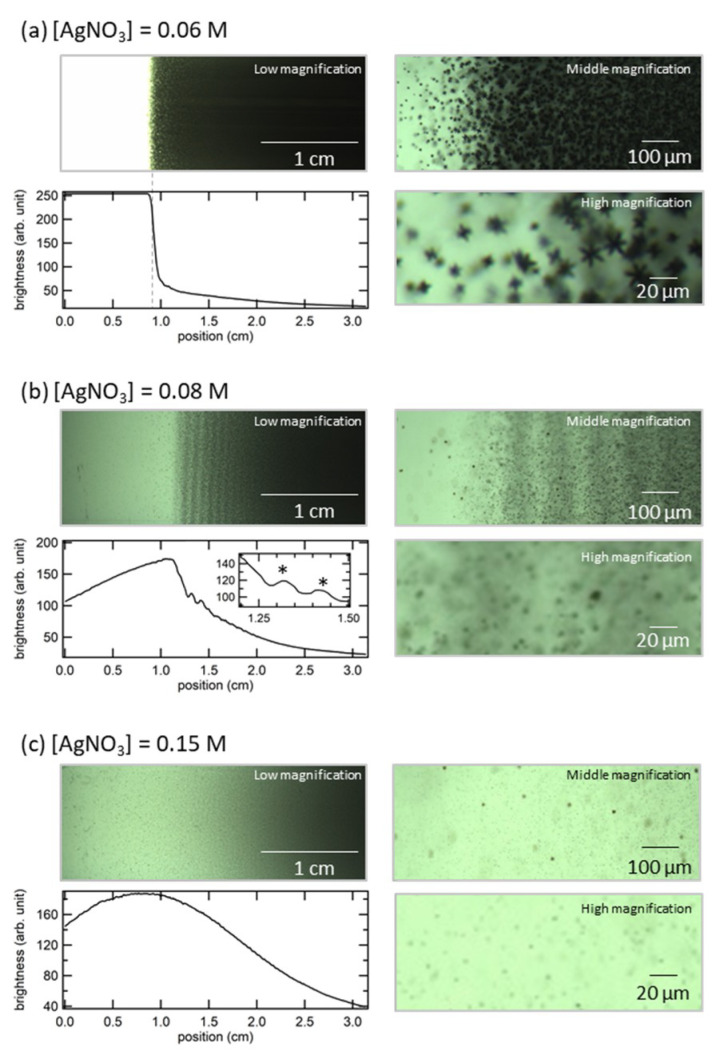
Microscopic images and brightness line profiles for three different experimental conditions. [NaCl] was fixed at 0.01 M, and [AgNO_3_] was (**a**) 0.06 M, (**b**) 0.08 M, and (**c**) 0.15 M. Asterisks in (**b**) indicate the position of band.

To compare the details of the precipitated zones, we observed the reaction front under three different conditions using optical microscopy. At lower [AgNO_3_] conditions, a sharp reaction front can be seen at the bottom of the precipitated zone (Figure 2a), which is clear in the image at low magnification. The brightness line profile of the low-magnifi cation image also exhibited the presence of a sharp reaction front that was divided into precipitated and non-precipitated zones. The middle magnification image showed a gradient in the particle density near the reaction front, where the particle density sharply decreased at the reaction front. Furthermore, it can be noted that the particle size was also changed. Particles more than 20 μm in diameter were formed in the dense region, whereas the particles existing in the front region were approximately 10 μm, as shown in the image with high magnification. The decrease in the particle density and particle size strongly indicates that the reaction front was clearly observed because of the rapid suppression of the particle growth reaction in front of a certain boundary position. From the images, we can also see that the particles in both regions are star-shaped, although they are different in size. This is in agreement with a previous study showing that AgCl microcrystals several tens of micrometers in size form a star-shaped structure. [33]. At higher [AgNO_3_] conditions (Figure 2c), there was no noticeable edge on the precipitated zone, as shown in Figure 1. The absence of a clear reaction front was also confirmed by the brightness line profile, where only a gradual intensity change was observed. Magnified images show the formation of small particles with a size of a few micrometers. In contrast to the formation of star-shaped particles at the reaction front for [AgNO_3_] = 0.06 M, the particle at [AgNO_3_] = 0.15 M appears to have a non-star-shaped structure, similar to a sphere or cubic, which are structures that form in the process of forming star-shaped AgCl [33]. In the middle [AgNO_3_] condition (Figure 2b), a periodic precipitated zone formed near the reaction front. The brightness line profile also indicated the presence of the periodic formation of the precipitated zone. The magnified images show the formation of small particles similar to those at [AgNO_3_] = 0.15 M, along with a band which resembled a shadow.

**Figure 3:**
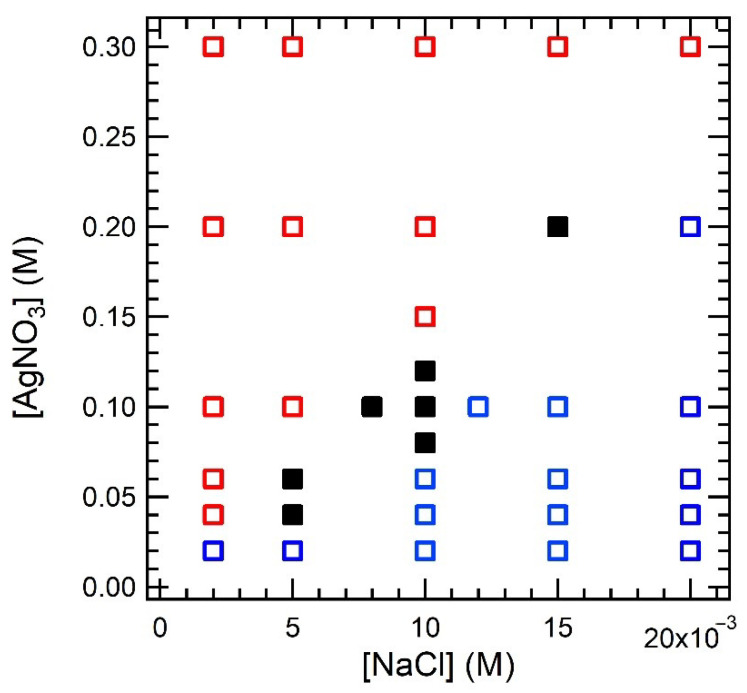
Phase diagram of the precipitation mode of AgCl as a function of [NaCl] and [AgNO_3_]. Red open square: continuous precipitation with a gradual edge, black closed square: periodic precipitation, and blue open square: continuous precipitation with a sharp edge.

**Figure 4:**
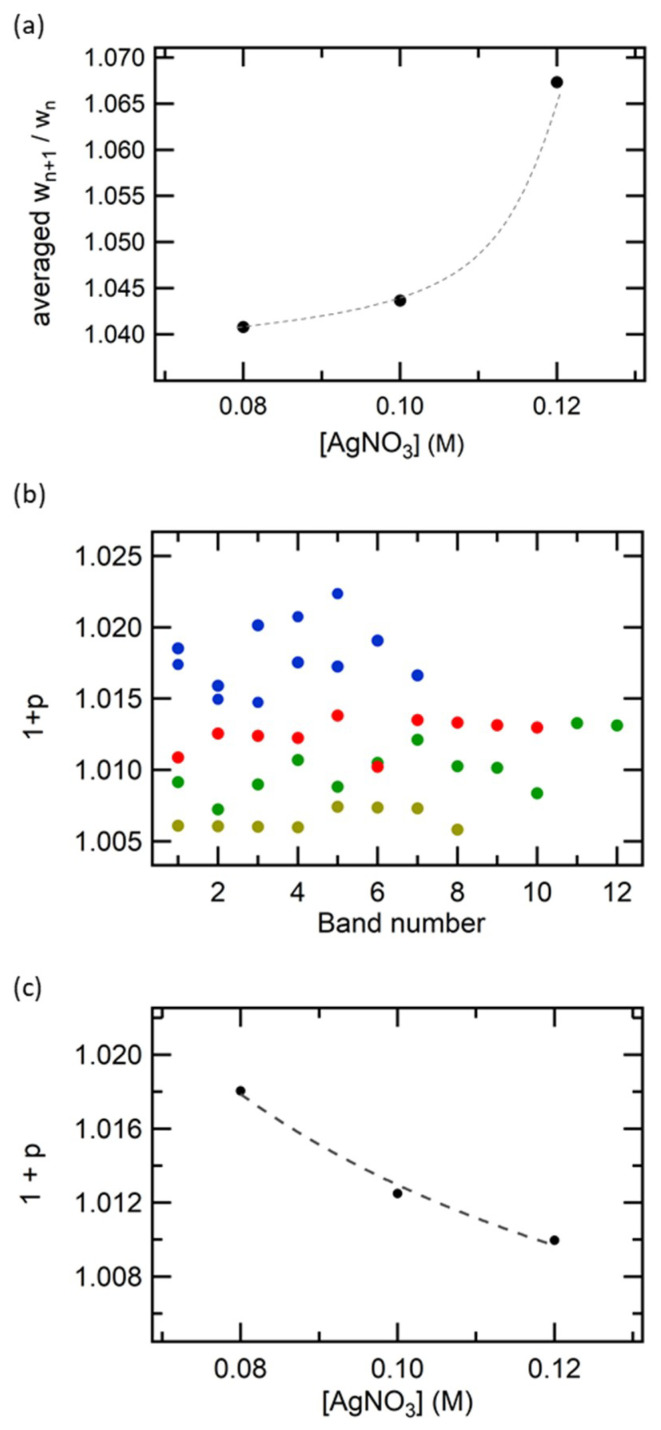
(**a**) Averaged *w_n+_*_1_/*w_n_* as the function of [AgNO_3_]. (**b**) Variation in (1 + *p*) as the function of the band number. Blue: [NaCl] = 0.01 M, [AgNO_3_] = 0.08 M; red: [NaCl] = 0.01 M, [AgNO_3_] = 0.10 M; green: [NaCl] = 0.01 M, [AgNO_3_] = 0.12 M; and brown: [NaCl] = 0.015 M, [AgNO_3_] = 0.20 M. (**c**) Variation in (1 + *p*) as the function of [AgNO_3_], where the dashed line depicts the best-fit to the Matalon–Packter law (Equation (2)).

From the above microscopic observations, it was found that there were at least three types: (i) continuous precipitation with a sharp edge at lower [AgNO_3_], (ii) periodic precipitation in the middle [AgNO_3_] and (iii) continuous precipitation with a gradual edge at higher [AgNO_3_]. We conducted the same experiments under various [NaCl] conditions to acquire a phase diagram to categorize the above three types (Figure 3). The periodic precipitation was observed only at [AgNO_3_] = 0.04–0.20 M, below and above which only continuous precipitation was observed. The formation of periodic bands at only limited concentration conditions is a common feature of the Liesegang phenomenon [16]. Therefore, the current pattern could be formed under the same mechanism as the Liesegang phenomena. To verify whether *w_n+_*_1_/*w_n_* > 1 (regular type) or *w_n+_*_1_/*w_n_* < 1 (negative type) and whether the periodicity follows the spacing law, we performed an analysis of the obtained periodic structures. As shown in Figure 4a, all the periodic structures observed in the present condition exhibited *w_n+_*_1_/*w_n_* > 1, indicating that the pattern of AgCl in a 1D system is not a revert but a regular-type. The present results indicate that AgCl is a regular type, not only in 2D systems [31] but also in 1D systems. As for the reason for the formation of regular-type patterns in 2D AgCl, a previous paper proposed that the formation of concentration gradients is different for 1D and 2D systems. However, our current results confirmed that AgCl forms a regular-type periodic pattern for both 1D and 2D systems. Although it is not clear why AgCl forms a regular-type pattern, unlike AgBr and AgI, it is possible that preferential adsorption, which is the formation mechanism of the revert-type, is less likely to occur in the AgCl system. We have further analyzed the pattern geometry based on the spacing law for the observed regular-type pattern as shown in Figure 4b, where 1 + *p* was roughly constant with respect to the band number. Furthermore, the value of 1 + *p* appears to be dependent on both [AgNO_3_] and [NaCl], in which the value of 1 + *p* was plotted as a function of [AgNO_3_] at [NaCl] = 0.010 M, as shown in Figure 4c. As can be seen clearly, 1 + *p* gradually decreased with the increase in [AgNO_3_], which is consistent with the expectation from the Matalon–Packter law. Fitting the data with Equation (2) shows good agreement, indicating that the periodicity observed in the 1D NaCl precipitation pattern satisfies the spacing law and thus the Liesegang mechanism.

## 4. Conclusions

In this study, we observed the periodic precipitation reaction of AgCl in a 1D system to confirm whether the difference in the concentration gradient is responsible for the regular-type AgCl pattern in a 2D system, as previously reported. Experiments under various [NaCl] and [AgNO_3_] conditions demonstrated three types of precipitation modes: continuous precipitation with a sharp front, periodic precipitation and continuous precipitation with a gradual front. This difference in the precipitation mode was also confirmed to be accompanied by differences in the sizes and shapes of the precipitated particles. Larger and star-shaped particles were formed for continuous precipitation with a sharp front, whereas smaller and non-star-shaped particles were formed for the other two modes. With respect to periodic precipitation, *w_n+_*_1_/*w_n_* was found to be larger than unity for any condition. Furthermore, *p* changed according to the Matalon–Packter law. These results strongly confirm that AgCl in a 1D system forms the regular-type Liesegang pattern, which is the same tendency observed in the 2D system. Therefore, unlike AgBr and AgI which show the revert-type Liesegang pattern, we can conclude that AgCl forms regular-type Liesegang patterns regardless of the dimension of diffusion.

## Data Availability

The data presented in this study are available on request from the corresponding author.
